# Influenza infection presenting with pulmonary congestion: influenza myocarditis

**DOI:** 10.1002/rcr2.352

**Published:** 2018-07-30

**Authors:** Masahiro Yamasaki, Takenori Okada, Yoji Sumimoto, Noboru Hattori

**Affiliations:** ^1^ Department of Respiratory Disease Hiroshima Red Cross Hospital & Atomic‐bomb Survivors Hospital Hiroshima Japan; ^2^ Department of Cardiology Hiroshima Red Cross Hospital & Atomic‐bomb Survivors Hospital Hiroshima Japan; ^3^ Department of Cardiovascular Medicine, Graduate School of Biomedical and Health Sciences Hiroshima University Hiroshima Japan; ^4^ Department of Molecular and Internal Medicine, Graduate School of Biomedical and Health Sciences Hiroshima University Hiroshima Japan

**Keywords:** Chest CT, influenza, influenza myocarditis, myocarditis, pulmonary congestion

## Abstract

Influenza myocarditis is a rare but life‐threatening complication of influenza infection. Pneumonitis is a well‐known complication of influenza infection, and chest computed tomography (CT) is useful for diagnosing pneumonitis. In addition, myocarditis should be considered in cases of pulmonary congestion observed on chest CT.

## Abstract 2

Influenza myocarditis is a rare but life‐threatening complication of influenza infection. The primary treatment for influenza myocarditis is supportive intervention. We report a case of influenza myocarditis diagnosed from pulmonary congestion observed on chest computed tomography (CT), and that recovered after intensive care using percutaneous cardiopulmonary support (PCPS) and intra‐aortic balloon pumping (IABP).

## Graphical abstract

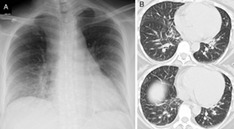

## Clinical Image

Influenza myocarditis is a rare but life‐threatening complication of influenza infection 1. The primary treatment for influenza myocarditis is supportive intervention. We report a case of influenza myocarditis diagnosed from pulmonary congestion observed on chest computed tomography (CT) and that recovered after intensive care using percutaneous cardiopulmonary support (PCPS) and intra‐aortic balloon pumping (IABP).

**Figure 1:**
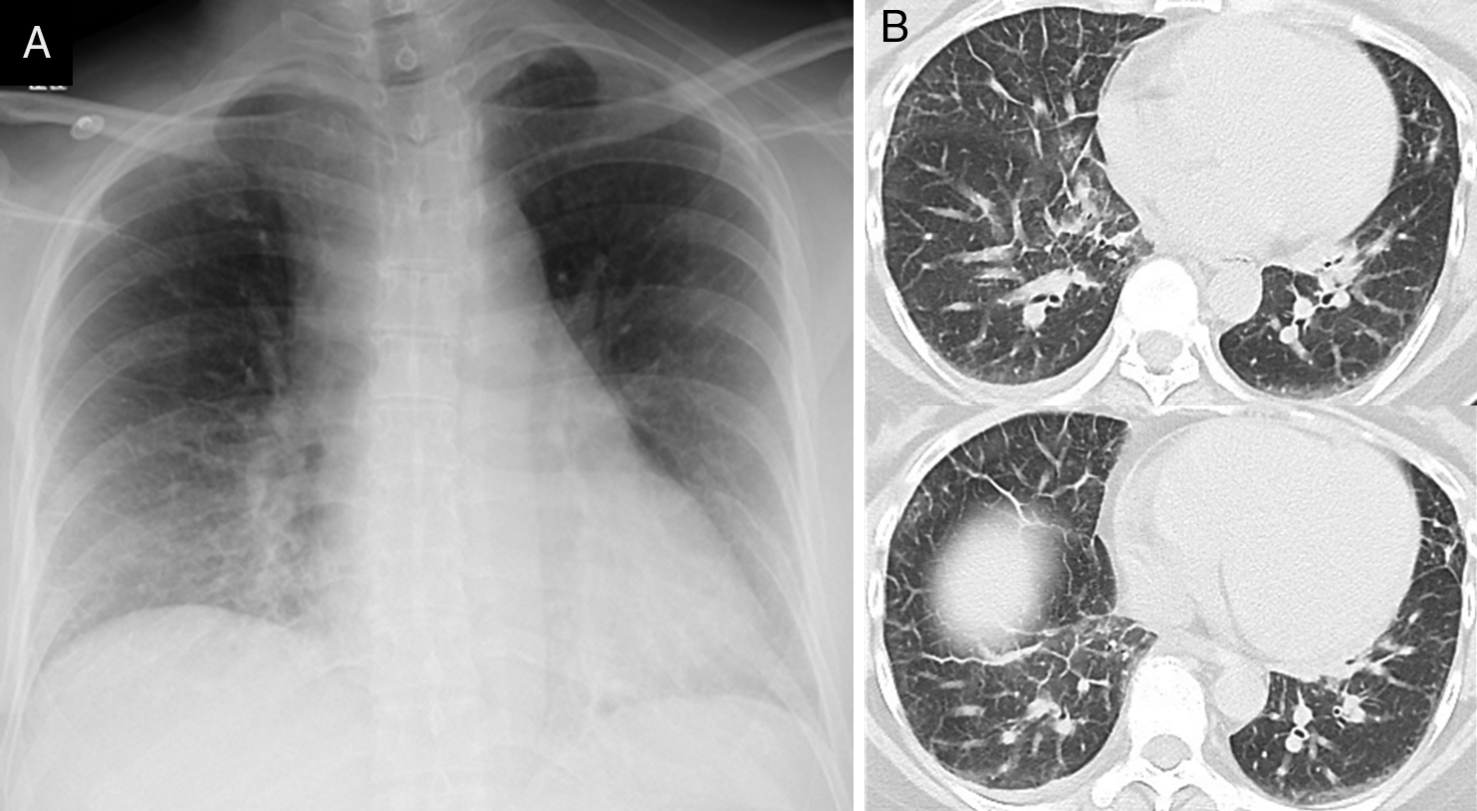
(A) Chest radiograph obtained on admission showing faint infiltration in the lower lung fields. (B) Computed tomography scan showing interlobular septal thickening and peribronchovascular interstitial thickening.

**Figure 2:**
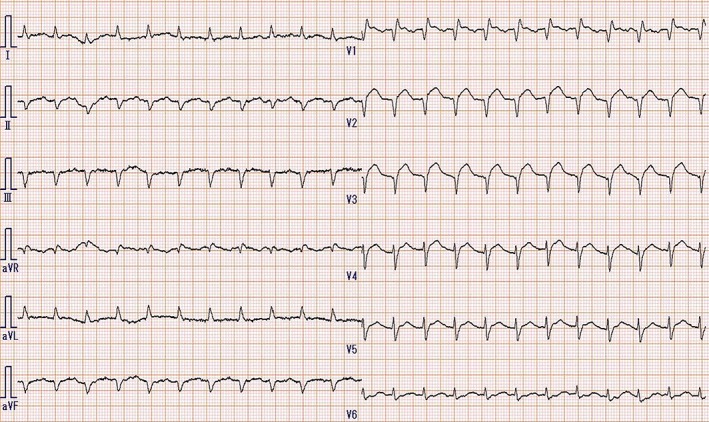
Electrocardiogram showing a QS pattern in leads II, III, aVF, and V1–3 and an ST‐segment elevation in leads V1–3.

A 48‐year‐old woman with fever, productive cough, dyspnoea, general fatigue, and nausea was referred to our hospital after consultation with a general physician for high‐grade fever 3 days previously. She was diagnosed with influenza A infection using a test kit for rapid diagnosis. Zanamivir, an inhalable powder, was administered. No particular personal medical history was reported, except for a diagnosis of Kawasaki disease during her childhood. She had high fever, tachycardia, tachypnoea, and fine crackles in her lower chest upon auscultation. Heart sounds demonstrated no remarkable changes. Chest radiography showed faint infiltration in the lower lung fields (Fig. 1A). A diagnosis of pneumonitis was suspected; unexpectedly, chest CT revealed pulmonary congestion (Fig. 1B). Electrocardiography showed a QS pattern in leads II, III, aVF, and V1–3 and an ST‐segment elevation in leads V1–3 (Fig. 2), and her serum creatine phosphokinase: 642 U/L (normal range: 45–163 U/L), troponin I: >50 ng/mL (<0.04 ng/mL), and B‐type natriuretic peptide: 528.5 pg./mL (<18.4 pg./mL) were increased. Echocardiography demonstrated severe diffuse hypokinesis of the left ventricle, and the ejection fraction was reduced to 23.8%. Finally, she was diagnosed with influenza myocarditis and was transferred to a specialized treatment centre. She was administered intravenous dobutamine, diuretics, and peramivir. Thereafter, she developed fulminant myocarditis. Fortunately, she recovered after 8 days of intensive care using PCPS and IABP. Meanwhile, cardiac catheterization was conducted, no coronary artery disease was detected, and myocarditis was confirmed by endomyocardial biopsy.

The present case shows two important clinical observations. First, influenza patients presenting with symptoms of dyspnoea may be diagnosed with myocarditis. Myocarditis is diagnosed on the basis of a combination of compatible symptoms, abnormal electrocardiography results, elevated cardiac enzyme levels, and cardiac dysfunction 2. Acute myocardial infarction should also be considered in differential diagnoses 3. Occasionally, coronary angiography and/or cardiac scintigraphy examinations are required.

Second, pulmonary congestion observed on chest CT might be a useful finding for diagnosis of myocarditis in a patient with influenza infection. Pulmonary congestion is a sign of cardiac dysfunction. Chest CT is useful for the diagnosis of pneumonitis 4, which is a well‐known complication of influenza infection. In addition, myocarditis should also be considered in cases presenting with severe respiratory symptoms, especially pulmonary congestion observed on chest CT.

The primary treatment for influenza myocarditis is supportive intervention. In case of severe dysfunction, using PCPS and/or IABP improves outcomes 5. Early and correct diagnosis of myocarditis leads to saving the lives of patients.

In conclusion, myocarditis should be considered in influenza cases presenting with severe respiratory symptoms, especially pulmonary congestion observed on chest CT.

## Disclosure Statement

Appropriate written informed consent was obtained for publication of this case report and accompanying images.
